# Determining the relationship between dietary iodine intake, urinary iodine excretion and thyroid functions in people with type 2 diabetes mellitus

**DOI:** 10.20945/2359-3997000000233

**Published:** 2020-03-30

**Authors:** Rahime Evra Karakaya, Mendane Saka, Didem Ozdemir

**Affiliations:** 1 Ankara Yildirim Beyazit University Faculty of Health Sciences Department of Nutrition and Dietetics Ankara Turkey Ankara Yildirim Beyazit University, Faculty of Health Sciences, Department of Nutrition and Dietetics, Ankara, Turkey; 2 Baskent University Faculty of Health Sciences Department of Nutrition and Dietetics Ankara Turkey Baskent University, Faculty of Health Sciences, Department of Nutrition and Dietetics, Ankara, Turkey; 3 Ankara Yildirim Beyazit University Faculty of Medicine Department of Endocrinology and Metabolism Ankara Turkey Ankara Yildirim Beyazit University, Faculty of Medicine, Department of Endocrinology and Metabolism, Ankara, Turkey

**Keywords:** Type 2 diabetes mellitus, iodine, iodine deficiency, thyroid disorders

## Abstract

**Objective:**

Type 2 diabetes mellitus (T2DM) is a worldwide health problem, and medical nutrition therapy is essential for improving the quality of life of patients with type 2 diabetes. Salt restriction may lead to iodine deficiency in these patients. Moreover, type 2 diabetes can be an indirect reason for thyroid disorders. This study was conducted to determine the relationship between dietary iodine intake, urinary iodine excretion and thyroid functions in people with T2DM.

**Materials and methods:**

Iodine nutritional status was determined by a one day 24-h dietary recall and food-frequency questionnaire. Iodine status was detemined by urinary iodine excretion with morning urine sample.

**Results:**

Iodine intake according to one day 24-h dietary recall was lower in T2DM patients [94.8 (76.0-112.0) μg] than people in control group [137.1 (123.1-165.4) μg] (p < 0.05). Iodine intake determined by food-frequency questionnaire rich in iodine was lower in T2DM patients [93.1 (84.4-113.9) μg] than people in control group [140.2 (125.1-166.1) μg] (p < 0.05). Mild iodine deficiency was found in %38.8 of diabetic and %55.1 of healthy individuals (p < 0.05). No significant relationship was found between urinary iodine excretion and thyroid function tests in groups (p > 0.05). However, the relationship between dietary iodine excretion and urinary iodine intake in the diabetic group was lower than in the control group (p < 0.05).

**Conclusion:**

With this respect, the results showed that while planning medical nutrition therapy for diabetic individuals, the risk of iodine deficiency should be considered.

## INTRODUCTION

Type 2 diabetes mellitus (T2DM) is a chronic metabolic disease that requires continuous medical care in which the body can not benefit sufficiently from carbohydrates, fats and proteins due to insulin deficiency or impairment of insulin action. Nowadays diabetes has become a health problem all over the world due to its frequency and complications. While the number of people with diabetes in the world was 415 million in 2015, it is predicted that this number will reach 642 million by 2040 with an increase of 55% (1). The main reasons for this increase are population growth, aging, and lifestyle changes brought about by urbanization, resulting in increased obesity and physical inactivity (2).

The goal of diabetes treatment is to prevent microvascular and macrovascular complications and control cardiovascular risk factors along with lowering blood glucose to normal levels. The first and main approach in all types of diabetes that should be continued during the illness is patient education, medical nutrition therapy (MNT) and exercise. MNT is the most important part of education for diabetes treatment and management (3). The goals of MNT are to provide metabolic control, prevent chronic complications of diabetes, determine the nutritional needs of the individual by taking the personal and cultural characteristics into consideration, and to achieve self-management skills in various situations (e.g. exercise, hypoglycemia, acute illness etc.) (4).

Iodine, a micronutrient, is an important component of the thyroid hormones required for the normal growth and development of the brain and nervous system and homeostasis (5). All diseases caused by inadequate iodine intake or impaired iodine metabolism are called “Iodine Deficiency Disorders (IDD)” (6). The optimal method to determine iodine deficiency is to assess the amount of urinary iodine. Inadequate iodine intake is usually associated with low iodine content in the soil, and therefore, low iodine content in the nutrients consumed. In addition, lower seafood consumption results in inadequate iodine intake (7). The adverse effect of iodine deficiency on the human body is the reduction of the formation of thyroid hormones. Inadequate iodine intake is the most important cause of goitre. Iodine deficiency may lead to goitre, hypothyroidism, mental retardation, infertility, lack of physical performance and increase in some types of thyroid cancer in adulthood (6).

Diabetes and thyroid diseases are endocrine diseases that interact each other (8). Studies determining the pathophysiological relationship between diabetes and thyroid function support that it is comprised of biochemical, genetic and hormonal dysfunctions (9,10). T2DM patients were found to have lower urinary iodine excretion than non-T2DM patients. Also, urinary iodine was negatively associated with glucose, insulin and insulin resistance (11). Adequate iodine intake is important in these patients as in the general population.

In this study, we aimed to evaluate dietary iodine intake and urinary iodine excretion in individuals with T2DM. Also, we investigated the possible association between iodine status and thyroid functions in these patients.

## MATERIALS AND METHODS

### Subjects

This study was conducted with 49 patients with T2DM and 49 healthy subjects aged 18-64 years were observed in our outpatient clinic between March-June 2017. Preliminary interviews were conducted with the patients and their suitability for the study was checked. T2DM patients receiving MNT and/or oral antidiabetics were included. Diabetic macrovascular and/or microvascular complications were determined as exlusion criteria. Indviduals who were not on any diet program were included in control group. All individuals with chronic diseases such as cancer, chronic liver disease, chronic renal failure, psychological disorders, thyroid diseases were excluded. Also, pregnant-lactating women, patients who are taking thyroid medication or who took it last year, who use medicines that might affect thyroid metabolism (steroids, amiodarone, lithium, etc.), who are taking iodine-containing vitamin supplements, who took iodine-containing drug in the last 6 months, who have poor blood glucose regulation (HbA1c > 8%), and those with iodine restriction were excluded from the study.

## DESIGN

A questionnaire form consisting of three sections (sociodemographic characteristics, information about diabetes, nutritional habits) was applied to participants with face-to-face interview method. One-day (24 h) dietary recall was taken to assess the intake of energy and macro and micro nutrients. Nutrients were analyzed by using “Computer Aided Nutrition Program, Nutrition Package Information Systems Program (BeBiS)” developed for Turkey (12). Turkomp (13) and BEBIS databases were used to determine the iodine content of foods. In addition, a form consisting of information about iodine intake (family history of goiter disease, iodised salt consumption status, storage conditions, duration and amount of iodised salt intake and etc.), frequency of consumption of 14 iodine rich foods (milk, yoghurt, cheese, egg, offal, oily fish, shellfish, other seafood, green leafy vegetables, other vegetables, potato, citrus, other fruits) and 3 goitrogenic foods (radish, turnip juice, cabbage) was applied. Anthropometric measurements of individuals (body weight, height, waist and hip circumference) were taken by a trained researcher. Body mass index (BMI) was calculated by the formula [(body weight (kg) / (height (m)^2^)] evaluated according to WHO criteria (14).

### Laboratory assays

Fasting blood glucose (FBG), glycosylated hemoglobin (HBA1c) and insulin levels were measured in the blood samples taken after 12 hours of fasting. Insulin resistance (HOMA-IR) was calculated by fasting insulin (μU/mL) x fasting plasma glucose (mg/dL)/22.5 formula (15). Thyroid functions were assessed by measuring plasma Thyrotrophin hormone (TSH), free triiodothyronine (fT3), free thyroxine (fT4), Antithyroglobulin** (**Anti Tg), and Anti-thyroid peroxidase (Anti TPO) values. The first urine samples were taken in the morning to determine urinary iodine excretion. Iodine status was determined by urinary concentration and evaluated according to criteria defined by WHO (16) (Table 1).

**Table 1:** Iodine nutrition assessment based on median iodine concentrations

Urinary iodine (μg/L)	Iodine intake	Iodine status
<20	Insufficient	Severe iodine deficiency
20-49	Insufficient	Moderate iodine deficiency
50-99	Insufficient	Mild iodine deficiency
100-199	Adequate	Adequate iodine nutrition
200-299	Above requirements	May pose a slight risk of more than adequate iodine intake in these populations
≥ 300	Excessive	Risk of adverse health consequences (iodine-induced hyperthyroidism, autoimmune thyroid disease)

### Statistical analysis

SPSS (IBM SPSS Statistics 20) package program was used to evaluate the data obtained from study and to prepare the tables. A square analysis was performed for the qualitative variables. The indepedent sample-t test (t-table value) was used to compare two independent groups of parametric methods with normal values for quantitative variables, which are shown as mean ± standard deviation (± SD). For quantitative variables, the Mann-Whitney U test (Z table value) was used to compare two independent groups as the values were not normally distributed, which are shown as median [Q1-Q3]. The correlation of two quantitative variables with normal distribution is examined by using “Pearson” and the correlation coefficient of “Spearman” is used as a result of having at least one quantitative variable with non-normal distribution. The significance level of all statistical analysis was accepted as p < 0.05 (17).

## RESULTS

The median age of the diabetic patients was 56.0 (53.0-61.0) years and higher than the control group [50.0 (49.0-55.0)] (p < 0.05). The mean duration of diabetes was 8.86 ± 7.32 years. 12.2% diabetic patients received only MNT, and 87.8% received MNT and oral antidiabetic treatment. Prevalence of subjects making regular physical activity was higher in diabetic group compared to healthy group (55.1% vs. 32.7%, p < 0.05) (unshown data).

Median BMI was significantly higher in diabetic group [30.7 (26.6-34.9)] than control group 27.4 (24.4-31.9) (p = 0.011). Biochemical parameters of the individuals demonstrated that the diabetic group had a significantly higher FBG value than the control group (p = 0.001). In addition, diabetic patients’ HbA1c and HOMA-IR levels were significantly higher than control group (p < 0.001 for each). There was no significant relationship between urinary iodine and plasma TSH, fT3, fT4, Anti Tg and Anti TPO values in patients with diabetes and control group. Similar results were obtained when further analysis was applied for women and men.

According to 24 h dietary recall, dietary idoine intake was 91.8% in diabetic and 69.4% in healthy individuals (p = 0.011) (Table 3). Similarly, diabetic men had significantly higher rate of inadequate iodine intake than healthy men (90.5% vs. 55.6%) (p = 0.013), while the difference in diabetic women and healthy women was not significant (p = 0.100). The daily intake of iodine determined by food frequency questionnaire including foods rich in iodine was inadequate in 95.9% of diabetic and 61.2% of the control groups (p < 0.001). Considering the results obtained from this questionnaire, inadequate iodine intake was higher in both diabetic women and men compared to healthy women and men, respectively (p = 0.002 and p = 0.003 respectively). In iodine status, there was a significant difference between diabetic patients and healthy controls determined by iodine excretion (p < 0.001). The significance was also valid for women and men (p < 0.001 for each). Moderate iodine deficiency was observed in 49.0% of diabetic patients whereas moderate iodine deficiency wasn’t detected in control group. Mild iodine deficiency was found in 38.8% of the diabetic individuals and in 55.1% of the control group. Urinary iodine excretion level was normal in 8.2% of the diabetic group and 42.9% of the control group.

**Table 3:** Grouping individuals’ dietary iodine intake and urinary iodine excretion according to adequate level

	Women					Men					All cases			

	Diabetic group (n: 28)		Control group (n: 31)			Diabetic group (n: 21)		Control group (n: 18)			Diabetic group (n: 49)		Control group (n: 49)	

	n	%	n	%		n	%	n	%		n	%	n	%
**Iodine intake (24 h dietary recall), µg/d**														
Inadequate (< 150)	26	92.9	24	77.4		19	90.5	10	55.6		45	91.8	34	69.4
Adequate (≥ 150)	2	7.1	7	22.6		2	9.5	8	44.4		4	8.2	15	30.6
	χ^2^ = 2.712, p = 0.100				χ^2^ = 6.199, p =** 0.013**					χ^2^ = 6.529, p = **0.011**				
**Iodine intake (FFQ), µg/d**														
Inadequate (< 150)	27	96.4	20	64.5		20	95.2	10	55.6		47	95.9	30	61.2
Adequate (≥ 150)	1	3.6	11	35.5		1	4.8	8	44.4		2	4.1	19	38.8
	χ^2^ = 9.247, p = **0.002**				χ^2^ = 8.598, p = **0.003**					χ^2^ =15.515, p = **0.000**				
**Urinary iodine excretion, µg/L**														
Severe iodine deficiency	2	7.1	-	-		-	-	-	-		2	4.1	-	-
Moderate iodine deficiency	12	42.9	-	-		12	57.1	-	-		24	49.0	-	-
Mild iodine deficiency	10	35.7	15	48.4		9	42.9	12	66.7		19	38.8	27	55.1
Optimal	4	14.3	15	48.4		-	-	6	33.3		4	8.2	21	42.9
Above requirements	-	-	1	3.2		-	-	-	-		-	-	1	2.0
	χ^2^ = 22.273, p = **0.000**				χ^2^ = 18.306, p = **0.000**					χ^2^ = 39.951, p = **0.000**				

FFQ: food frequency questionnaire.

A correlation analysis was made to identify the possible association between urinary iodine excretion and biochemical findings and dietary iodine intake in diabetic patients and healthy controls. There was no significant relationship between urinary iodine excretion and FBG, HbA1c, insulin, HOMA-IR, thyroid functions and thyroid autoantibodies in diabetic and healthy group (Table 4).

**Table 4:** Relationship between urinary iodine excretion and biochemical findings

	Urinary iodine excretion, µg/L											

	Women				Men				All cases			

	Diabetic group (n: 28)		Control group (n: 31)		Diabetic group (n: 21)		Control group (n: 18)		Diabetic group (n: 49)		Control group (n: 49)	

	r	p	r	p	r	p	r	p	r	p	r	p
FBG, mg/dL	0.011	0.956	-0.003	0.988	0.097	0.676	0.186	0.460	0.054	0.713	0.041	0.782
HbA1c, %	-0.279	0.151	0.036	0.846	0.170	0.463	0.163	0.518	-0.079	0.589	0.067	0.649
Insulin, mIU/mL	-0.137	0.488	0.071	0.705	-0.279	0.220	0.088	0.729	-0.142	0.330	-0.005	0.974
HOMA-IR, mmol/L	-0.130	0.509	0.034	0.856	-0.245	0.284	0.100	0.693	-0.137	0.347	0.042	0.772
TSH, uIU/mL	-0.032	0.870	-0.196	0.291	-0.115	0.620	0.070	0.782	0.028	0.850	-0.046	0.753
fT3, pg/mL	0.174	0.375	-0.211	0.254	-0.378	0.091	-0.447	0.063	-0.089	0.541	-0.217	0.134
fT4, ng/mL	-0.254	0.192	0.056	0.766	0.052	0.822	-0.324	0.190	-0.176	0.227	-0.011	0.939
Anti-Tg, IU/mL	0.004	0.983	-0.225	0.224	0.429	0.053	0.121	0.633	0.144	0.324	-0.108	0.462
Anti-TPO, IU/mL	0.012	0.951	-0.132	0.479	0.368	0.101	-0.071	0.779	0.184	0.182	-0.003	0.982

FBG: fasting blood glucose; HOMA-IR: Homeostasis Model Assessment – Insulin Resistance; TSH: Thyrotrophin; fT3: free triiodothyronin; fT4: free thyroxine; Anti-Tg: antithyroglobulin; Anti-TPO: antithyroid peroxidase.

A positive relationship was found between urinary iodine excretion and dietary iodine intake with 24 h dietary recall in diabetic and control groups (p < 0.001 and p = 0.001, respectively). There was a positive relationship between urinary iodine excretion and iodine intake with food frequency form in diabetic and control groups (p = 0.001 and p < 0.001, respectively) (Table 5). These associations were also observed when patients and control groups were subgrouped as women and men.

**Table 5:** Relationship between urinary iodine excretion and dietary iodine intake

	Urinary iodine excretion, µg/L											

	Women				Men				All cases			

	Diabetic group (n: 28)		Control group (n: 31)		Diabetic group (n: 21)		Control group (n: 18)		Diabetic group (n: 49)		Control group (n: 49)	

	r	p	r	p	r	p	r	p	r	p	r	p
Iodine intake (24 h dietary recall), µg/d	0.669	**0.000**	0.520	**0.003**	0.340	0.131	0.534	**0.023**	0.505	**0.000**	0.466	**0.001**
Iodine intake (FFQ), µg/d	0.569	**0.002**	0.625	**0.000**	0.430	0.052	0.478	**0.045**	0.458	**0.001**	0.522	**0.000**

FFQ: food frequency questionnaire.

## DISCUSSION

Metabolic control can be achieved via MNT with drug and exercise treatment in diabetic individuals. MNT is generally based on the individual’s diet to reduce total and saturated fat, to limit the salt to reduce sodium consumption, to increase the consumption of fiber and to provide a metabolic control with moderate weight loss. Especially, the restriction on salt consumption is important because of the high blood pressure risk which is frequently encountered in these patients (18). There may be differences in daily iodine intake owing to salt restriction during the treatment. It is thought that risk of iodine deficiency is increased with salt restriction in individuals (19). At the same time, inadequate iodine intake in these individuals may cause impairment of thyroid function (17).

Iodine deficiency is a common problem in developing countries and is thought to affect about 2 billion people around the world. As a result of iodine deficiency, conditions such as goiter, hypothyroidism, impaired mental functions, decreased fertility, hyperthyroidism and cancer can be encountered in adult individuals. The most effective method for detecting the iodine status is to determine the urinary iodine excretion as it is a sensitive indicator of changes in the amount of dietary iodine intake (20). In a study with Type 1 diabetes mellitus (T1DM) and T2DM subjects, it was found that the median urinary iodine excretion of T2DM individuals was 200.0 (21.0-411.0) μg/L, and 30% of them had excessive iodine excretion. Excessive iodine excretion was thought to be associated with osmotic diuresis. In T1DM patients, the median urinary iodine excretion was determined as 150 (12.0-393.0) μg/L and iodine deficiency was detected in 16% of them. It was thought that iodine deficiency may be associated with thyroid dysfunction due to thyroid autoimmunity, and diabetic individuals may be more susceptible to iodine deficiency disorders (8). Another study reported that low urinary iodine excretion in diabetic individuals was not a diabetic condition and was indicative of iodine deficiency due to low iodine intake in the community (21). In this study, the median urinary iodine excretion of diabetic subjects was significantly lower than control group [49.6 (36.3-57.0) μg/L vs. 91.5 (76.1-121.2) μg/L, p < 0.05] (unshown data). According to the classification of urinary iodine excretion, it was determined that diabetic individuals had lower urinary iodine excretion than the control group in general, also mild iodine deficiency was found in control group. These results suggest that iodine deficiency is a common health problem, thus diabetic individuals may be at higher risk.

Food frequency form with iodine rich foods and 24-hour dietary recall questionnaire are applied to determine dietary iodine intake. In a study, a 53-item food frequency form containing iodine-rich foods and a 4-day dietary recall form were used to determine dietary iodine intake (22). In this study, iodine intake in diabetic patients was significantly lower than the control group according to both 24-hour dietary recall form and food frequency questionnaire rich in iodine foods. This could be explained by caloric restriction of T2DM patients. Also, salt restriction due to MNT therapy may have resulted in less iodised salt intake in these patients.

The iodine deficiency in diabetic individuals may lead to thyroid dysfunctions. In a cohort study of T2DM subjects, a positive relationship was found between urinary iodine/creatinine ratio and TSH (11). In this study, we excluded individuals with thyroid disease, and diabetic and control group had normal thyroid functions. There was no significant relationship between urinary iodine and plasma TSH, fT3, fT4, Anti Tg and Anti TPO values in patients with diabetes and control group. Also, no significant relationship between urinary iodine excretion and thyroid functions was found in groups.

Determining dietary iodine intake with dietary recall and food frequency form is an optimal method in order to assess urinary iodine excretion (23). The food frequency form only includes iodine-rich foods, suggesting that it may be better correlated with urinary iodine when daily feces excretion with 10 μg of iodine is considered (22). In this study, a positive relationship was found between urinary iodine excretion and iodine intake with 24-h dietary recall in diabetic and control groups. There was also a positive relationship between urinary iodine excretion and iodine intake with food frequency in both groups. According to these results, dietary iodine intake is considered to be a good indicator of urinary iodine excretion.

### Strength and limitations

The strength of this study is that to our knowledge, this is the first study evaluating the dietary iodine intake, urinary iodine excretion and thyroid functions in people with T2DM. The limitation of this study is that the dietary iodine intake was declared by the participants which may not reflect the accurate iodine intake.

In conclusion, adequate dietary iodine intake of diabetic individuals is important for reducing iodine deficiency disorders and enhancing thyroid functions. Considering the risk of iodine deficiency while planning nutritional therapies may contribute to increasing the quality of T2DM patients’ lives.

**Table 2:** Mean ± SD and median [Q1-Q3] values of individuals’ BMI values and biochemical findings

	Women		p	Men		p	All cases		p
		
	Diabetic group (n: 28)	Control group (n: 31)		Diabetic group (n: 21)	Control group (n: 18)		Diabetic group (n: 49)	Control group (n: 49)	
		
	x̄ ± SS/Median	x̄ ± SS/Median		x̄ ± SS/Median	x̄ ± SS/Median		x̄ ± SS/Median	x̄ ± SS/Median	
BMI, kg/m^2^	34.0 [28.1-35.7]	30.0 [25.4-34.2]	**0.040 **	28.74±3.54	26.10±3.64	**0.028 **	30.7 [26.6-34.9]	27.4 [24.4-31.9]	**0.011 **
FBG, mg/dL	134.0 [119.0-149.3]	94.0 [83.0-98.0]	**0.000**	139.14±28.95	92.44±7.01	**0.000**	134.0 [118.5-150.5]	94.0 [84.5-98.0]	**0.000**
HbA1c, %	7.2 [6.6-7.4]	5.4 [5.1-5.6]	**0.000**	7.04±0.80	5.31±0.28	**0.000**	7.2 [6.5-7.5]	5.4 [5.2-5.6]	**0.000**
HOMA-IR, mmol/L	3.7 [2.8-5.6]	1.8 [1.4-2.1]	**0.000**	3.3 [2.9-4.5]	1.4 [1.5-2.1]	**0.000**	3.5 [2.8-5.2]	1.8 [1.4-2.1]	**0.000**
TSH, mIU/mL	1.8 [1.2-2.8]	1.8 [1.5-3.0]	0.590	1.37 ± 0.62	1.39±0.53	0.898	1.5 [0.9-2.1]	1.7 [1.3-2.3]	0.453
fT3, pg/mL	2.84±0.28	2.94±0.45	0.316	3.20±0.31	3.29±0.34	0.410	2.99±0.34	3.07±0.44	0.362
fT4, ng/mL	1.31±0.18	1.24±0.22	0.208	1.29±0.14	1.23±0.14	0.213	1.30±0.16	1.24±0.20	0.090
Anti-Tg, IU/mL	21.1 [17.4-29.3]	16.4 [12.4-25.2]	0.078	21.1 [16.3-28.2]	20.1 [16.1-23.0]	0.406	21.1 [17.0-28.4]	18.8 [12.5-24.7]	0.052
Anti-TPO, IU/mL	17.6 [13.9-28.5]	16.1 [12.5-20.5]	0.242	15.7 [11.9-22.0]	14.1 [11.9-16.5]	0.455	16.2 [13.1-25.3]	15.4 [12.4-20.2]	0.206

BMI: body mass index; FBG: fasting blood glucose; HOMA-IR: Homeostasis Model Assessment – Insulin Resistance; TSH: thyrotrophin; fT3: free triiodothyronin; fT4: free thyroxine; Anti-Tg: antithyroglobulin; Anti-TPO: antithyroid peroxidase.
